# Acquiring Wearable Photoplethysmography Data in Daily Life: The PPG Diary Pilot Study ^†^

**DOI:** 10.3390/ecsa-7-08233

**Published:** 2020-11-14

**Authors:** Peter H. Charlton, Panicos Kyriacou, Jonathan Mant, Jordi Alastruey

**Affiliations:** 1 Primary Care Unit, Department of Public Health and Primary Care, University of Cambridge, Cambridge CB1 8RN, UK; 2 Research Centre for Biomedical Engineering, City, University of London, London EC1V 0HB, UK; 3 Department of Biomedical Engineering, School of Biomedical Engineering and Imaging Sciences, King’s College London, King’s Health Partners, London SE1 7EH, UK

**Keywords:** photoplethysmogram, cardiovascular, wearable sensors, pulse wave, smartwatches

## Abstract

The photoplethysmogram (PPG) signal is widely measured by smart watches and fitness bands for heart rate monitoring. New applications of the PPG are also emerging, such as to detect irregular heart rhythms, track infectious diseases, and monitor blood pressure. Consequently, datasets of PPG signals acquired in daily life are valuable for algorithm development. The aim of this pilot study was to assess the feasibility of acquiring PPG data in daily life. A single subject was asked to wear a wrist-worn PPG sensor six days a week for four weeks, and to keep a diary of daily activities. The sensor was worn for 75.0% of the time, signals were acquired for 60.6% of the time, and signal quality was high for 30.5% of the time. This small pilot study demonstrated the feasibility of acquiring PPG data during daily living. Key lessons were learnt for future studies: (i) devices which are waterproof and require charging less frequently may provide signals for a greater proportion of the time; (ii) data should either be stored on the device or streamed via a reliable connection to a second device for storage; (iii) it may be beneficial to acquire signals during the night or during periods of low activity to achieve high signal quality; and (iv) there are several promising areas for PPG algorithm development including the design of pulse wave analysis techniques to track changes in cardiovascular properties in daily life.

## 1 Introduction

The photoplethysmogram (PPG) signal is widely measured by smart watches and fitness bands. Whilst the PPG is primarily used for heart rate monitoring, the signal contains a wealth of additional information on the cardiovascular, respiratory and autonomic nervous systems. Consequently, the PPG may also be useful for a range of additional applications, such as detecting irregular heart rhythms [1], tracking infectious diseases [2], and monitoring blood pressure [3]. Research into signal processing techniques to analyse the PPG would benefit greatly from publicly available datasets of PPG signals acquired in daily life.

There are several challenges to acquiring PPG data in daily living. Firstly, it is difficult to record PPG signals acquired by a wearable. They must either be stored on a wearable device, or transferred to a second recording device. However, the memory capacity of wearables is often insufficient for prolonged PPG recordings, and Bluetooth connections can be unreliable. Secondly, a wearable needs to be sufficiently comfortable for prolonged use. Thirdly, it needs to be robust enough to the rigors of daily life, including being waterproof. Fourthly, the device needs to either have a long battery life, or be charged regularly and reliably. Finally, the wearable must provide the PPG signal rather than solely the derived numerics provided by many commercially available devices such as heart rate. Even wearables designed for use in research do not necessarily meet these requirements [4].

It is important to develop approaches to acquire PPG data in daily life as publicly available PPG datasets are of great value. They provide several benefits including: (i) acting as benchmark data with which to compare techniques developed by different researchers; (ii) facilitating exploratory, hypothesis-generating analyses which do not warrant novel data collection; (iii) making the field accessible to those who cannot acquire their own data; and (iv) aiding reproducibility. Indeed, in a recent review over a third of studies of PPG signal processing techniques used publicly available datasets [5]. Despite these benefits, there are very few publicly available PPG datasets acquired in daily life (the PPG-DaLiA dataset is a notable exception, acquired in close to real-life conditions [6]).

The aim of this study was to assess the feasibility of acquiring PPG data in daily life. A single participant was asked to wear a wearable device for four weeks, and keep a diary of their activities of daily living. The results of the study provide valuable insight into how best to acquire PPG data in daily life to aid future research. In addition, the dataset and analysis code are publicly available.

## 2 Methods

### 2.1 Study Participant

An adult volunteer participated in the study. The participant provided written informed consent to participate. The study was conducted in accordance with the Declaration of Helsinki, and was registered with the King’s College London Research Ethics Office (MRA-18/19-10495).

### 2.2 Data Collection

The volunteer was asked to wear a wrist-worn device to acquire PPG signals six days a week, for four weeks. The participant was also asked to record the times and nature of any daily activities, particularly those which may influence the PPG signal (e.g., typewriting, exercise, and sleeping). They were asked to wear the device for the whole day apart from: (i) when recharging the device (approx. 2–3 h per day); (ii) for one day each week for a break; (iii) during activities which could damage the device (such as showering or vigorous exercise); (iv) during any unhygienic activities (such as using the toilet); and (v) if they experienced discomfort or wanted a break.

**Figure 1:**
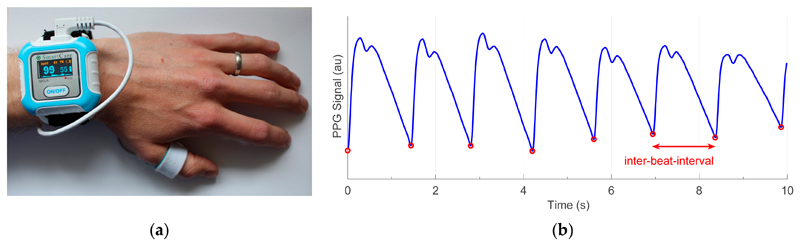
Acquiring photoplethysmography (PPG) signals in daily living: (**a**) the SmartCare wrist-worn pulse oximeter used to record the PPG at the thumb; (**b**) a sample PPG signal (in arbitrary units), with red circles indicating individual heartbeats.

The SmartCare wrist-worn pulse oximeter (SmartCare Analytics Ltd., London, UK) was used as shown in Figure 1. The device acquired red and infrared PPG signals at 100 Hz at the thumb. The data were streamed to a mobile phone via Bluetooth for storage. The participant was asked to re-connect the device and phone if the Bluetooth disconnected, and record the time of re-connection if possible.

### 2.3 Data Curation and Analysis

The data were manually reviewed to identify connection and disconnection times which were not noted by the participant, and to correct any misaligned times to within 5 s of the event.

**Figure 2:**
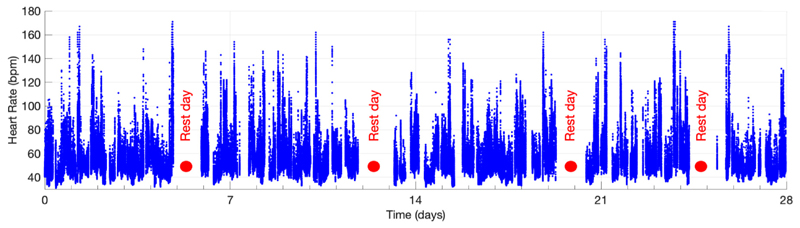
Data were collected from a single volunteer over a period of 28 days including four rest days. Data shown here are heart rates.

The infrared PPG signal was used for analysis. The PPG was band-pass filtered with –3dB cutoffs of 0.4 Hz and 16.8 Hz. Signal quality was assessed using an adaptation of the algorithm described in [7] and coded in PulseAnalyse (v.1.2beta) [8,9]. The heart rates shown in Figure 2 were provided by the device. Changes in PPG pulse wave shape during activities of daily living were analysed by: identifying individual heartbeats using the algorithm in [10]; calculating beat-to-beat heart rates; and median-filtering the heart rates.

## 3 Results and Discussion

**Figure 3:**
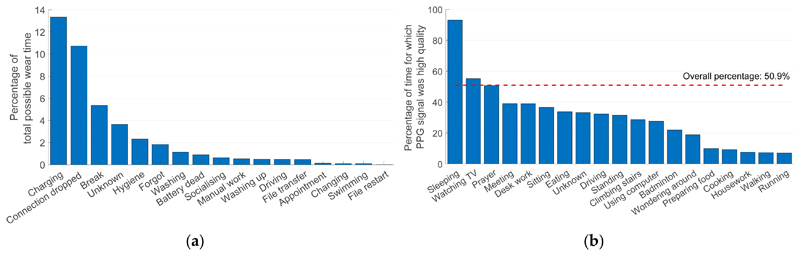
Data capture and data quality: (**a**) reasons for data loss; (**b**) the data quality associated with different activities of daily living.

The young volunteer (a male in the 18–39 age range) participated for the full 28 days, and PPG data were collected throughout as shown in Figure 2. The study endpoints used to assess the feasibility of acquiring PPG data during daily living are provided in Table 1. The reasons for data loss and the data quality during different activities of daily living are shown in Figure 3.

### 3.1 Device Usage

The device was worn for 75.0% of the possible wear time (18.0 out of 24.0 days), as reported in Table 1. It was primarily removed for charging (13.4% of the time), as well as for activities involving water, for hygiene purposes, when having a break, and when the participant forgot to reattach the device (see Figure 3a). In this study, the sensor probe was located on the thumb and was not waterproof, so it often had to be temporarily removed. In the future, it may be beneficial to use a waterproof device which measures the PPG at the wrist to avoid the need to remove it during daily activities such as washing and using the toilet. These changes may also reduce the number of occasions on which participants forget to reattach the device after removing it.

### 3.2 Data Capture

A PPG signal was acquired for 60.6% of the possible wear time (349.3 h, *i.e.*, 14.6 days), as detailed in Table 1. PPG data were primarily lost due to dropped Bluetooth connections (accounting for 10.7% of the time, see Figure 3a). In total 241 Bluetooth disconnections (10.0 per day) were recorded, each requiring the user to manually reconnect the device. This demonstrates the need for reliable wireless connectivity if data are to be streamed from the wearable to a data logging device, and highlights the potential benefit of storing the data on the wearable either permanently, or in a buffer to mitigate against temporary disconnections, to avoid data loss due to connectivity issues.

### 3.3 Signal Quality

Signal quality was high for 30.5% of the time (Table 1) and 50.9% of the time for which a signal was recorded (Figure 3b). The proportion of the time for which high quality signals were acquired varied greatly between activities as shown in Figure 3b. It was highest during sleep (93.1% of the time), followed by sedentary activities such as watching TV (55.2%). It was particularly low during exercise (7.4% during walking and 7.0% during running) or activities involving hand movement such as preparing food (9.9%), cooking (9.3%) and housework (7.6%). This indicates the potential benefit of acquiring PPG signals during sleep when movement is minimal and the ambient light level is low. It also indicates that PPG signal quality appears to be related to movement, and therefore the signal should preferably be acquired at rest. Indeed, some devices acquire a PPG signal during low activity levels to increase signal quality [11]. Finally, this shows the importance of algorithms to extract parameters from the PPG accurately, even in the presence of motion artifacts [12].

### 3.4 Potential Utility for Pulse Wave Analysis

**Figure 4:**
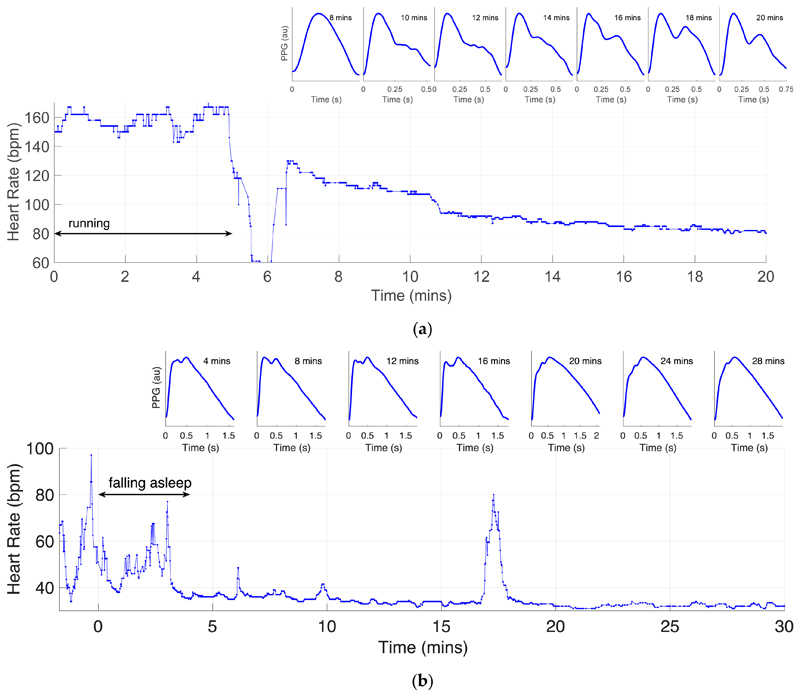
Changes in PPG pulse wave shape during activities of daily living: (**a**) during recovery from exercise; (**b**) whilst falling sleep.

The PPG signals acquired by the device often exhibited the distinct systolic and diastolic peaks on each pulse wave which are associated with young, healthy subjects (as shown in Figure 1b). This indicates that the device may be suitable for pulse wave analysis studies. Pulse wave shape changed considerably during activities of daily living. For instance, Figure 4a shows the changes as heart rate reduced during recovery from exercise, with the amplitude of the diastolic peak increasing over time. Figure 4b shows the changes in the first 30 min of sleep, with the amplitude of the systolic peak decreasing over time. These may indicate that cardiovascular properties such as arterial stiffness or blood pressure changed during the activities. These examples illustrate potential areas for exploratory analyses to develop techniques to assess cardiovascular state from the PPG in daily life.

### 3.5 Limitations and Future Work

The key limitations to this study are that data were acquired from a single participant, using a single device. Therefore, the findings may not be generalisable. Different people may conduct different activities during daily life, and in different ways. In addition, devices for acquiring PPG signals are being developed continuously, and new devices may provide even better performance than the current device. Activities of daily living were self-reported in this study: future studies may consider the use of body-worn cameras to obtain gold-standard labels of activities.

This study provides several directions for future research. Future research should assess whether the findings of this study are consistent between participants and between devices. In particular, it is important to assess whether PPG signal quality is closely related to the level of movement. If so, then devices could be designed to only record PPG signals during low movement levels, thus extending battery life and reducing memory consumption.

## 4 Conclusions

This small pilot study demonstrated the feasibility of acquiring PPG data during daily living. Key lessons were learnt for future studies: (i) devices which are waterproof and require charging less frequently may acquire signals for a greater proportion of the time; (ii) data should either be stored on the device or streamed via a reliable connection to a second device for storage; (iii) it may be beneficial to acquire signals during the night or during periods of low activity to achieve high signal quality; and (iv) there are several promising areas for PPG algorithm development including the design of pulse wave analysis techniques to track changes in cardiovascular properties in daily life. The dataset and analysis code are publicly available at [13], and further details are provided at: [14].

## Figures and Tables

**Table 1:** The data capture and data quality results, shown as the time and percentage of the maximum possible time.

Endpoint	Time, Hours (%)
**Possible wear time**: the time for which the participant was asked to wear the device	576.8 (100.0)
**Wear time**: the time for which the participant noted they wore the device	432.3 (75.0)
**Signal time**: the time for which a PPG signal was acquired	349.3 (60.6)
**High quality signal time**: the time for which a high quality signal was acquired	176.1 (30.5)
